# Supplementary material for: Understanding interactions between risk factors, and assessing the utility of the additive and multiplicative models through simulations
Source: PLoS One. 2021 Apr 26;16(4):e0250282. doi: 10.1371/journal.pone.0250282 (PMC8075235; doi:10.1371/journal.pone.0250282)
Supplement: S1 Fig — A-C: Simulation schemes for three generalized scenarios: synergism of causes (A), heterogeneity of causes (B) and a 5-factor threshold (C). The numbers are example frequencies, and frequencies in bold highlight the higher frequencies of the simulated risk factors (X and Z) associated with disease. For example, “Z: freq. 0.3” means that each simulated individual in the group had a 30% chance of being assigned the risk factor Z. The numbers in italics are the average frequency in the other group of simulated individuals; note that this will depend on the prevalence (which is adjusted in the scenarios in the split between cases and controls). “Components” (comp1 and comp2) were used as a strategy to obtain probabilistic risk factors. D: The relative risks for double risk (RR11) calculated from the simulation scenarios, with boxes summarizing 1,000 simulation runs with different risk factor frequencies. The observed RR11 are compared to the additive and multiplicative combinations of the relative risks for single risk (RR10 and RR01). Boxplots show median and quartiles for the simulations, but extreme values are omitted for clarity. Yellow arrows highlight where the median is visibly close to multiplicativity, while blue arrows do the same for additivity, for the two most extreme simulated prevalence rates. “M. threshold” means scenario C. E: Correlation coefficients between the risk factors X and Z. The relevant signal in each case is whether the median is negative, zero or positive, highlighted with a -, 0 or + symbol for the two most extreme simulated prevalence rates. We left out the highest prevalence results due to division-with-zero difficulties in the multiple threshold scenario. (PDF) [file pone.0250282.s001.pdf]

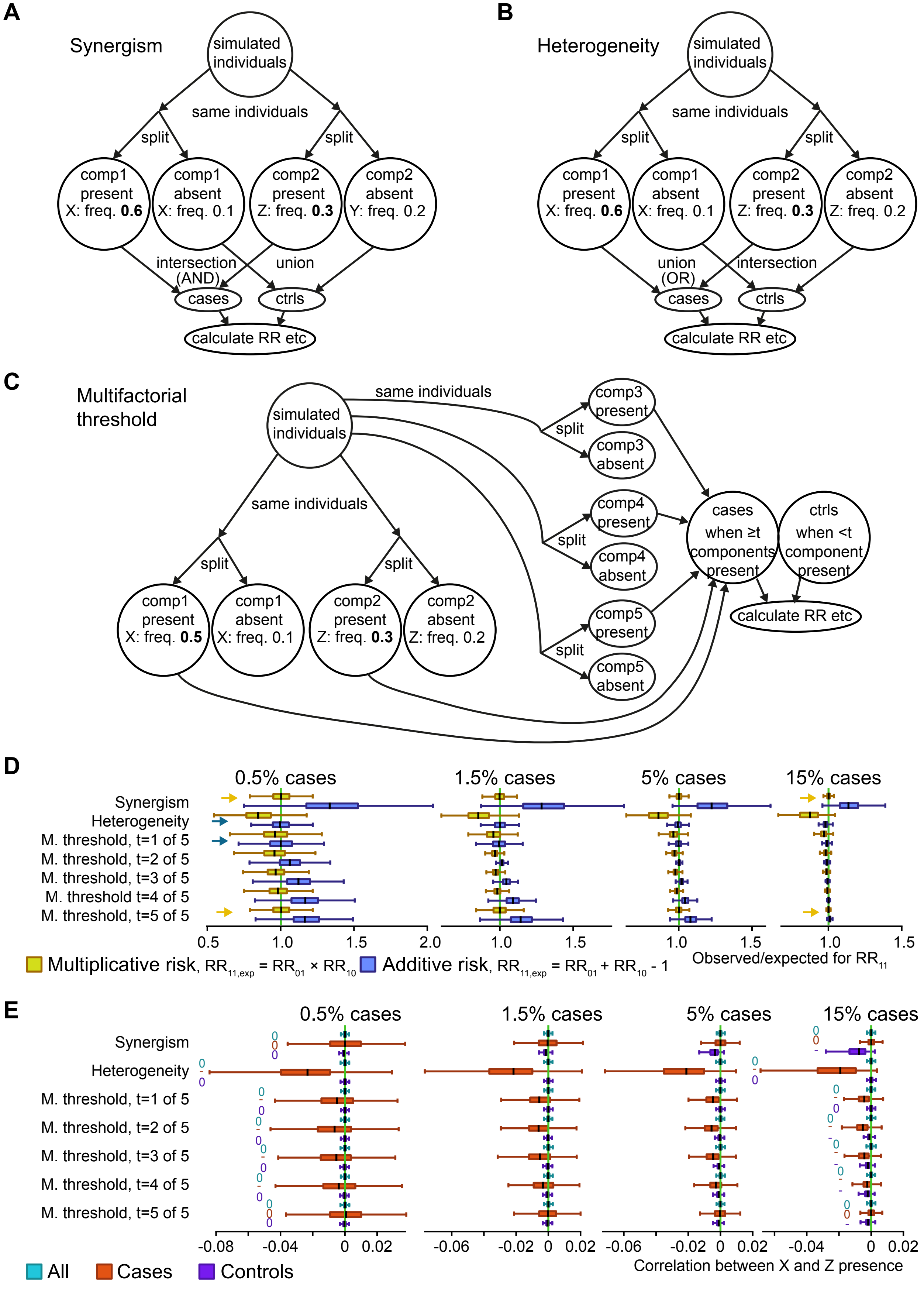

S1 Fig. Three simulated causal scenarios.

A-C: Simulation schemes for three generalized scenarios: synergism of causes (A), heterogeneity of causes (B) and a 5-factor threshold (C). The numbers are example frequencies, and frequencies in bold highlight the higher frequencies of the simulated risk factors (X and Z) associated with disease. For example, “Z: freq. 0.3” means that each simulated individual in the group had a 30% chance of being assigned the risk factor Z. The numbers in italics are the average frequency in the other group of simulated individuals; note that this will depend on the prevalence (which is adjusted in the scenarios in the split between cases and controls). “Components” (comp1 and comp2) were used as a strategy to obtain probabilistic risk factors.

D: The relative risks for double risk (RR_{11}) calculated from the simulation scenarios, with boxes summarizing 1,000 simulation runs with different risk factor frequencies. The observed RR_{11} are compared to the additive and multiplicative combinations of the relative risks for single risk (RR_{10} and RR_{01}). Boxplots show median and quartiles for the simulations, but extreme values are omitted for clarity. Yellow arrows highlight where the median is visibly close to multiplicativity, while blue arrows do the same for additivity, for the two most extreme simulated prevalence rates. “M. threshold” means scenario C.

E: Correlation coefficients between the risk factors X and Z. The relevant signal in each case is whether the median is negative, zero or positive, highlighted with a -, 0 or + symbol for the two most extreme simulated prevalence rates. We left out the highest prevalence results due to division-with-zero difficulties in the multiple threshold scenario.
