# Supplementary material for: Understanding interactions between risk factors, and assessing the utility of the additive and multiplicative models through simulations
Source: PLoS One. 2021 Apr 26;16(4):e0250282. doi: 10.1371/journal.pone.0250282 (PMC8075235; doi:10.1371/journal.pone.0250282)
Supplement: S2 Fig — The y axis is the signed square of the y axis in Fig 2A, otherwise this is the same plot, i.e. based on the scenario in Fig 1C. The red curve is the threshold fraction; this and fest make up the y axis. The notches in the box plots show bootstrapped 95% confidence intervals for the medians. The simulation had 0.5% cases and the rest controls, with a range ±0.04%. (PDF) [file pone.0250282.s002.pdf]

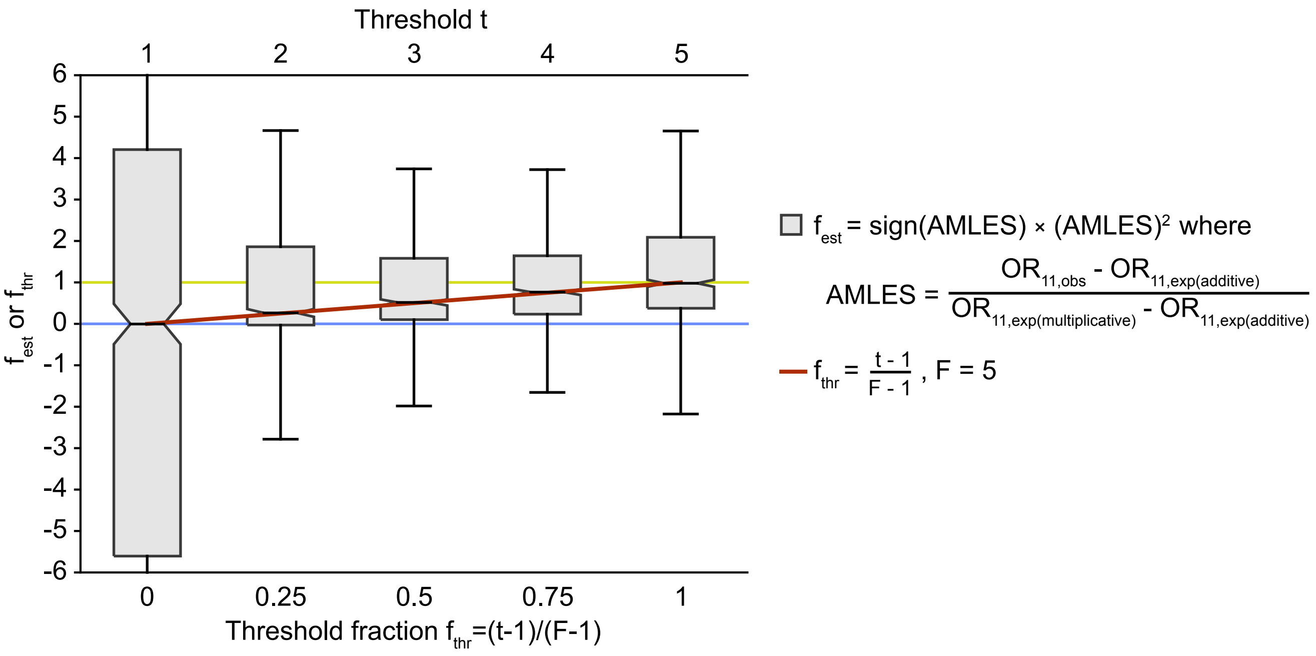

S2 Fig. Multifactorial threshold model with f_{est} metric. The y axis is the signed square of the y axis in Fig 2A, otherwise this is the same plot, i.e. based on the scenario in Fig 1C. The red curve is the threshold fraction; this and fest make up the y axis. Notched in the box plots show bootstrapped 95% confidence intervals for the medians. The simulation had 0.5% cases and the rest controls, with a range ±0.04%.
