# Supplementary material for: Understanding interactions between risk factors, and assessing the utility of the additive and multiplicative models through simulations
Source: PLoS One. 2021 Apr 26;16(4):e0250282. doi: 10.1371/journal.pone.0250282 (PMC8075235; doi:10.1371/journal.pone.0250282)
Supplement: S3 Fig — As in the scheme to the left, we simulated with (purple arrows) or without (green arrow) a step that reduced the number of controls to the same as the number of cases. As can be seen in the box plots above, this does not appear to have any effect on the results. (PDF) [file pone.0250282.s003.pdf]

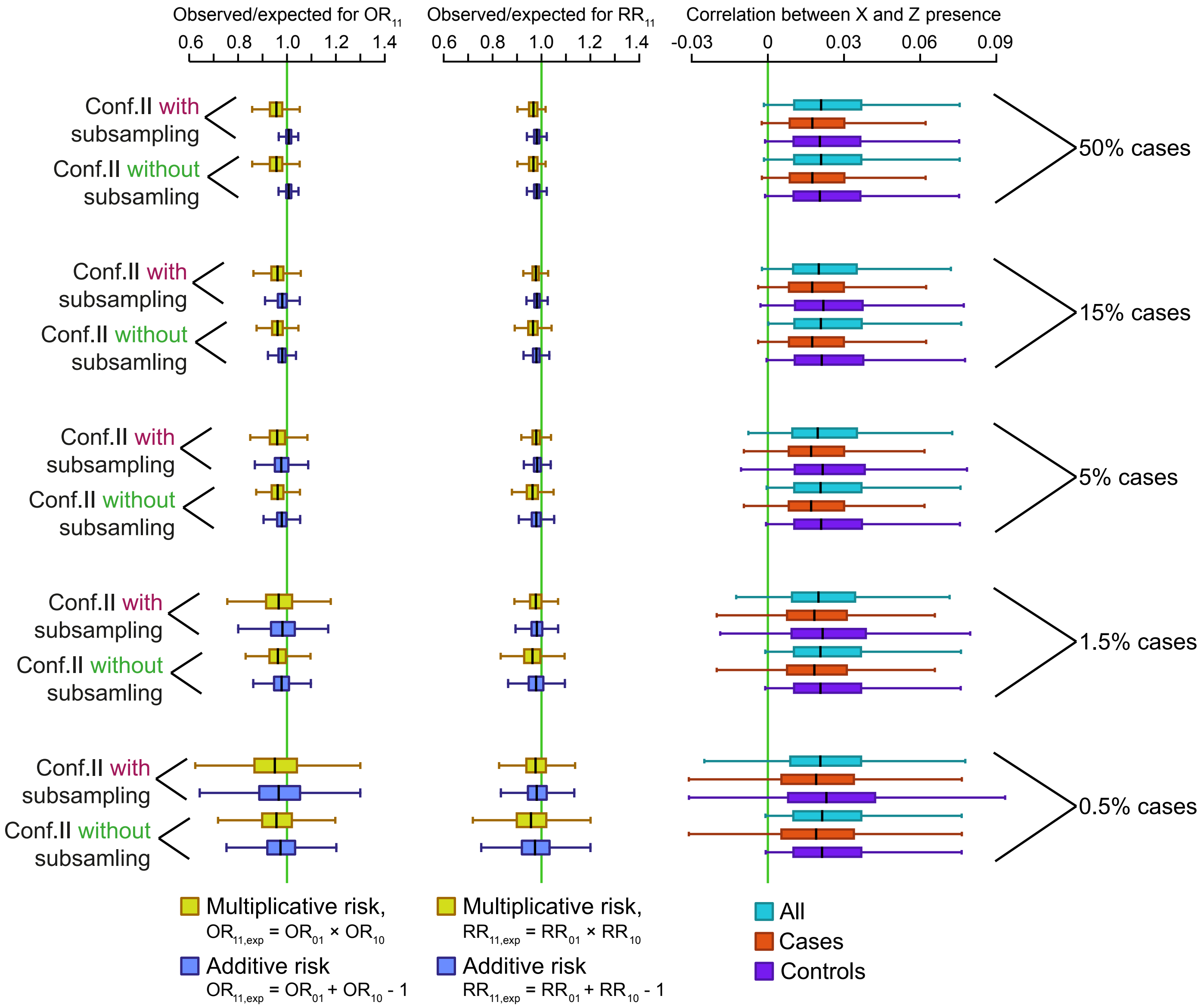

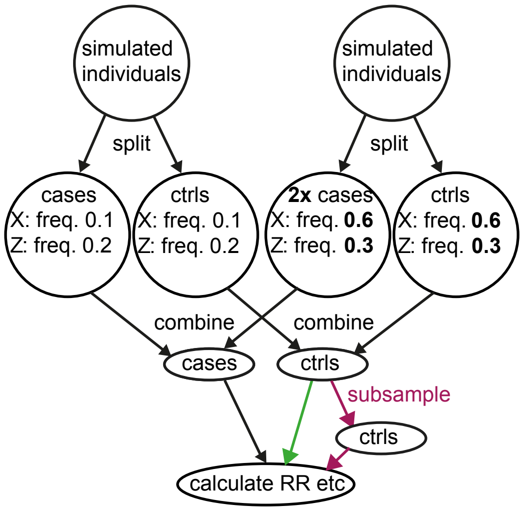

S3 Fig. No apparent difference from subsampling to equal number of controls and cases in Confounder II. As in the scheme to the left, we simulated with (purple arrows) or without (green arrow) a step that reduced the number of controls to the same as the number of cases. As can be seen in the box plots above, this does not appear to have any effect on the results.
