# Supplementary material for: Understanding interactions between risk factors, and assessing the utility of the additive and multiplicative models through simulations
Source: PLoS One. 2021 Apr 26;16(4):e0250282. doi: 10.1371/journal.pone.0250282 (PMC8075235; doi:10.1371/journal.pone.0250282)
Supplement: S4 Fig — A-B: Simulation for two schemes intended to produce additive effect. The numbers are example frequencies, and frequencies in bold highlight the higher frequencies of the simulated risk factors (X and Z) associated with disease. For example, “Z: freq. 0.3” means that each simulated individual in the group had a 30% chance of being assigned the risk factor Z. The numbers in italics are the average frequency in the other group of simulated individuals; note that this will depend on how many cases there are to controls, specifically, higher_freq × case_fraction + lower_freq × (1 –case_fraction). In each indicated circle the two risk factors have been added in an uncorrelated/independent manner. C-D: The relative risks (C) and odds ratios (D) for double risk (RR11 or OR11) calculated from the simulation schemes, with boxes summarizing 1,000 simulation runs with different risk factor frequencies. The observed RR11 were compared to the expected combinations of the relative risks for single risk (OR10 and OR01) in the additive and multiplicative models. Boxplots show median and quartiles for the simulations, but extreme values are omitted for clarity. Yellow arrows highlight where the median is visibly close to the null hypothesis for the multiplicative model, while blue arrows do the same for the additive model, for the two most extreme simulated trait prevalence rates. E: Correlation coefficients between the risk factors X and Z. The relevant signal in each case is whether the median is negative, zero or positive, highlighted with a -, 0 or + symbol for the two most extreme simulated prevalence rates. (PDF) [file pone.0250282.s004.pdf]

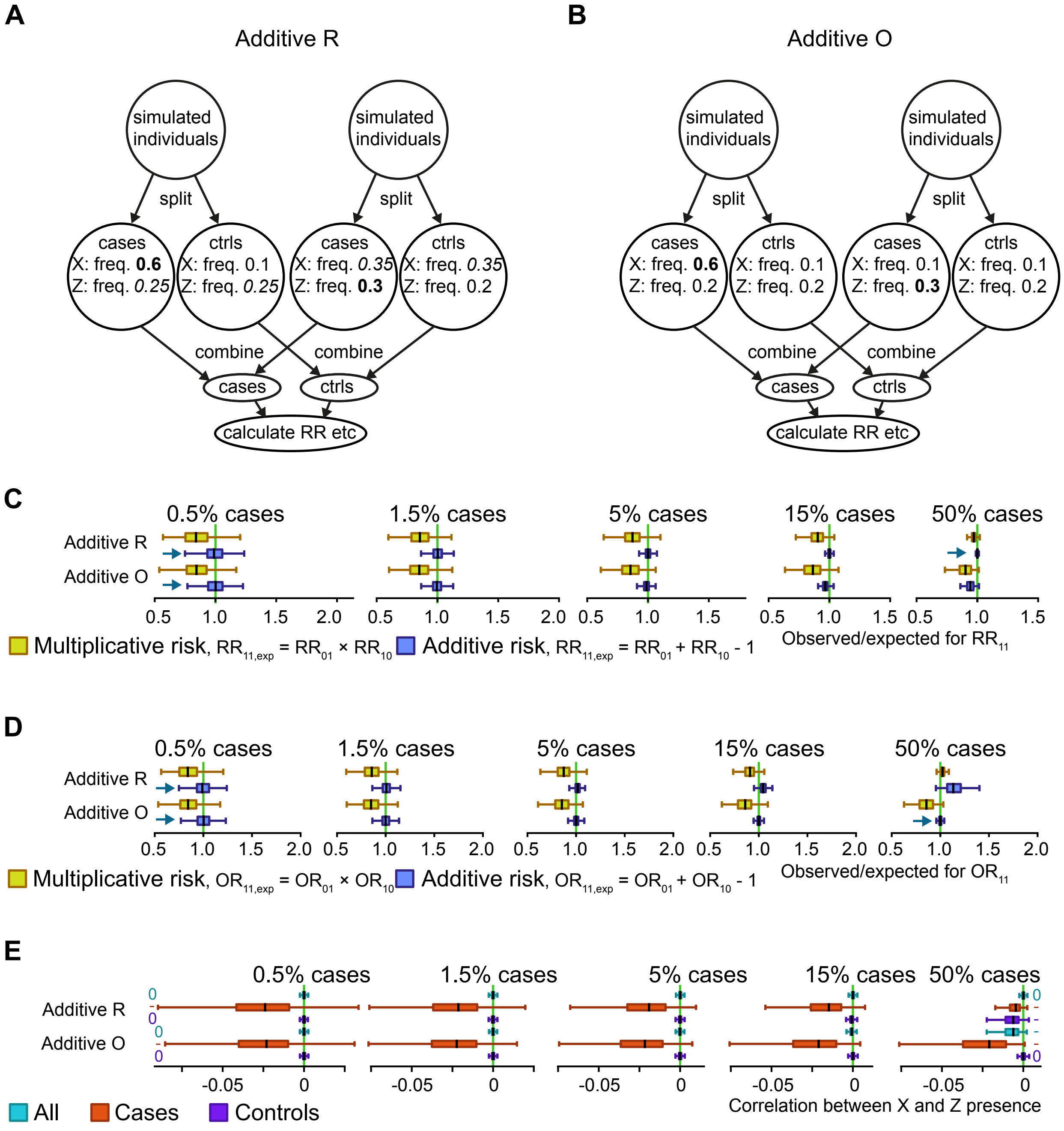

S4 Fig. Schemes providing additivity.

A-B: Simulation for two schemes intended to produce additive effect. The numbers are example frequencies, and frequencies in bold highlight the higher frequencies of the simulated risk factors (X and Z) associated with disease. For example, "Z: freq. 0.3" means that each simulated individual in the group had a 30% chance of being assigned the risk factor Z. The numbers in italics are the average frequency in the other group of simulated individuals; note that this will depend on how many cases there are to controls, specifically, higher_freq × case_fraction + lower_freq × (1 – case_fraction). In each indicated circle the two risk factors have been added in an uncorrelated/independent manner.

C-D: The relative risks (C) and odds ratios (D) for double risk (RR_{11} or OR_{11}) calculated from the simulation schemes, with boxes summarizing 1,000 simulation runs with different risk factor frequencies. The observed RR_{11} were compared to the expected combinations of the relative risks for single risk (OR_{10} and OR_{01}) in the additive and multiplicative models. Boxplots show median and quartiles for the simulations, but extreme values are omitted for clarity. Yellow arrows highlight where the median is visibly close to the null hypothesis for the multiplicative model, while blue arrows do the same for the additive model, for the two most extreme simulated trait prevalence rates.

E: Correlation coefficients between the risk factors X and Z. The relevant signal in each case is whether the median is negative, zero or positive, highlighted with a -, 0 or + symbol for the two most extreme simulated prevalence rates.
