# Supplementary material for: Understanding interactions between risk factors, and assessing the utility of the additive and multiplicative models through simulations
Source: PLoS One. 2021 Apr 26;16(4):e0250282. doi: 10.1371/journal.pone.0250282 (PMC8075235; doi:10.1371/journal.pone.0250282)
Supplement: S5 Fig — Correlation between HLA-DRB1 Shared Epitope (calculated as codominant) and other SNPs (calculated as dominant) in EIRA data within cases, control or all samples, for all non-HLA SNPs (A, C) or only known non-HLA risk SNPs (B). P-values come from 1-sample t-tests against zero. Regressing out sex and the first ten principal components gave essentially the same result (C) as without this step (A, B). (PDF) [file pone.0250282.s005.pdf]

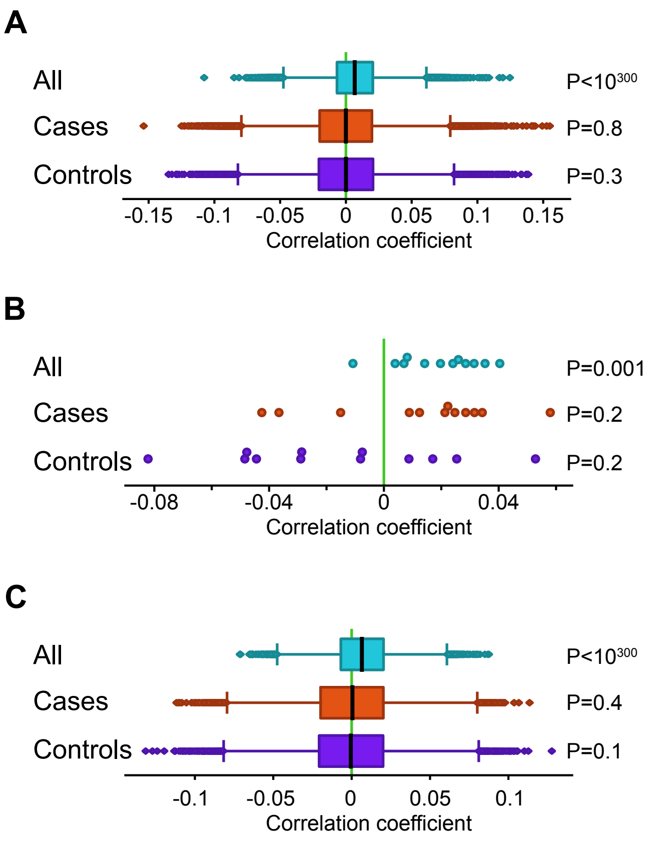

S5 Fig. Shared epitope correlation relationship. Correlation between HLA-DRB1 shared epitope (calculated as codominant) and other SNPs (calculated as dominant) in EIRA data within cases, control or all samples, for all non-HLA SNPs (A, C) or only known non-HLA risk SNPs (B). P-values come from 1-sample t-tests against zero. Regressing out sex and the first ten principal components gave essentially the same result (C) as without this step (A, B).
