# Supplementary material for: Understanding interactions between risk factors, and assessing the utility of the additive and multiplicative models through simulations
Source: PLoS One. 2021 Apr 26;16(4):e0250282. doi: 10.1371/journal.pone.0250282 (PMC8075235; doi:10.1371/journal.pone.0250282)
Supplement: S6 Fig — A-C: SE is HLA-DRB1 Shared Epitope, “other” are non-HLA risk SNPs for ACPA-positive RA. Unmod. or Unmodified is the EIRA data as it is, Rand1 shuffles, for each locus independently, within cases and shuffling within groups to match the independence part of the confounder scenario of Fig 3A (confounder I), Rand2 assigns individuals randomly into two groups and then in one group samples risk factor SE from controls and in the other group samples the other SNP risk factor from controls, to match S2B Fig (Additive O). We suspect that Rand2 unfortunately moderates odds ratios, meaning it could use a better replacement. Unmodified and Rand1 have similar variability (P = 0.04–0.8, Levene’s test, excluding (C) where calculations failed (NaN)). The middle of the scale for (C) is magnified to ease viewing. Blue arrows highlight where the median is visibly close to additivity, while yellow arrows do the same for multiplicativity. D: SNPs from Fig 4C and 4D, in varying numbers due to division-by-zero errors, with effect scaled between additive (blue line) and multiplicative (yellow line), as in Fig 2A. (PDF) [file pone.0250282.s006.pdf]

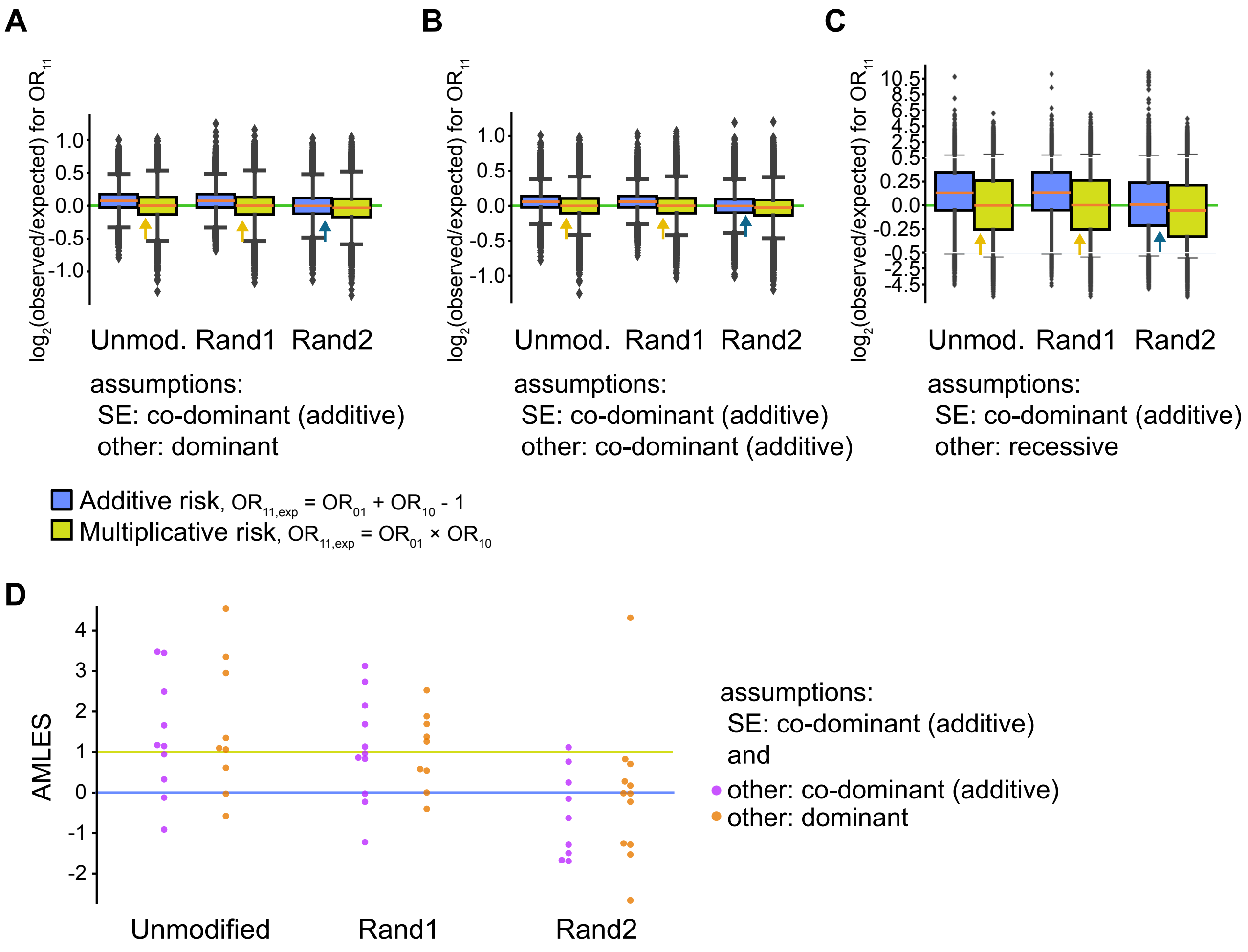

S6 Fig. Comparison of EIRA double risk odds ratios to two types of randomizations.

A-C: SE is *HLA-DRB1* Shared Epitope, "other" are non-HLA risk SNPs for ACPA-positive RA. Unmod. or Unmodified is the EIRA data as it is, Rand1 shuffles, for each locus independently, within cases and shuffling within groups to match the independence part of the confounder scenario of Fig 3A (Confounder I), Rand2 assigns individuals randomly into two groups and then in one group samples risk factor SE from controls and in the other group samples the other SNP risk factor from controls, to match S2B Fig (Additive O). We suspect that Rand2 unfortunately moderates odds ratios, meaning it could use a better replacement. Unmodified and Rand1 have similar variability (P=0.04-0.8, Levene's test, excluding (C) where calculations failed (NaN)). The middle of the scale for (C) is magnified to ease viewing. Blue arrows highlight where the median is visibly close to additivity, while yellow arrows do the same for multiplicativity.

D: SNPs from Figure 4C-D, in varying numbers due to division-by-zero errors, with effect scaled between additive (blue line) and multiplicative (yellow line), as in Fig 2A.
