# Supplementary material for: Understanding interactions between risk factors, and assessing the utility of the additive and multiplicative models through simulations
Source: PLoS One. 2021 Apr 26;16(4):e0250282. doi: 10.1371/journal.pone.0250282 (PMC8075235; doi:10.1371/journal.pone.0250282)
Supplement: Attachment — Submitted filename: Rebuttal_letter.docx [file pone.0250282.s008.docx]

Rebuttal letter, for " Understanding interactions between risk factors, and assessing the utility of the additive and multiplicative models through simulations" (PONE-D-20-24738R1).

On most points, the reviewer found the manuscript satisfactory. On one point, English, the reviewer did not. We reply to that below.

Additional Editor Comments (if provided):

As indicated by the reviewer, we suggest you thoroughly copyedit your manuscript for language usage, spelling, and grammar. If you do not know anyone who can help you do this, you may wish to consider employing a professional scientific editing service.

Upon resubmission, please provide the following:
- The name of the colleague or the details of the professional service that edited your manuscript
- A copy of your manuscript showing your changes by either highlighting them or using track changes
- A clean copy of the edited manuscript

Janet Ahlberg, who runs a professional editing service (Janet Ahlberg Firma, https://se.linkedin.com/in/janet-ahlberg-12240327, janetahlberg@yahoo.co.uk), has edited our manuscript. Two copies of our manuscript, with and without tracked changes, are included in the submission.

5. Is the manuscript presented in an intelligible fashion and written in standard English?

PLOS ONE does not copyedit accepted manuscripts, so the language in submitted articles must be clear, correct, and unambiguous. Any typographical or grammatical errors should be corrected at revision, so please note any specific errors here.

Reviewer #1: No

Response: See the comment at the bottom of this document.

6. Review Comments to the Author

Reviewer #1: The paper has greatly improved. I only strongly recommend a thorough language/grammar revision as many sentences added to the revised version can be improved

Response: The language usage, spelling, and grammar of our manuscript has been copy edited, by the authors and then by a professional scientific editing service (Janet Ahlberg, at Janet Ahlberg Firma). Almost every paragraph has been affected, by the around 500 changes to wording and formatting. We thank the reviewer (and editor) for pointing it the problem and letting us polish the manuscript.
